# Homologous NF-YC2 Subunit from *Arabidopsis* and Tobacco Is Activated by Photooxidative Stress and Induces Flowering

**DOI:** 10.3390/ijms13033458

**Published:** 2012-03-13

**Authors:** Dieter Hackenberg, Ulrich Keetman, Bernhard Grimm

**Affiliations:** Institute of Biology/Plant Physiology, Humboldt-Universität Berlin, Philippstr.13, Building 12, 10115 Berlin, Germany; E-Mails: dieter.hackenberg@biologie.hu-berlin.de (D.H.); keetman@gmx.de (U.K.)

**Keywords:** transcription factor, gene family, expression profile, photooxidative stress, flowering time

## Abstract

The transcription factor NF-Y consists of the three subunits A, B and C, which are encoded in *Arabidopsis* in large gene families. The multiplicity of the genes implies that NF-Y may act in diverse combinations of each subunit for the transcriptional control. We aimed to assign a function in stress response and plant development to NF-YC subunits by analyzing the expression of *NF-Y* genes and exploitation of *nf-y* mutants. Among the subunit family, *NF-YC2* showed the strongest inducibility towards oxidative stress, e.g. photodynamic, light, oxidative, heat and drought stress. A tobacco *NF-YC* homologous gene was found to be inducible by photooxidative stress generated by an accumulation of the tetrapyrrole metabolite, coproporphyrin. Despite the stress induction, an *Arabidopsis nf-yc2* mutant and *NF-YC2* overexpressors did not show phenotypical differences compared to wild-type seedlings in response to photooxidative stress. This can be explained by the compensatory potential of other members of the *NF-YC* family. However, *NF-YC2* overexpression leads to an early flowering phenotype that is correlated with increased *FLOWERING LOCUS T-*transcript levels. It is proposed that *NF-YC2* functions in floral induction and is a candidate gene among the NF-Y family for the transcriptional activation upon oxidative stress.

## 1. Introduction

Many promoters of eukaryotes contain the CCAAT–box motif, which is positioned between nucleotide −50 and −120, upstream of the transcription start site [1], and found also for multiple plant genes [1,2]. The CCAAT-box is recognized by the heterotrimeric transcription factor NF-Y (nuclear factor Y), which is also designated CCAAT-box-binding factor (CBF) or heme-activated proteins (HAP) 2, 3 and 5 [3–5]. The NF-Y-complex consists of three subunits A, B and C, which are characterized by conserved protein domains: The C-terminus of NF-YA, the central region of NF-YB, and the N-terminus of NF-YC show sequence identities of more than 70% in a stretch of 56, 90 and 84 amino acids among the NF-Y subunit homologs, respectively [6].

NF-YB and NF-YC contain also motifs of the histone 2A (H2A) and histone 2B (H2B) subfamilies and form a heterodimer at the interface of the histone-fold-motif (HFM) of each subunit [7,8]. Both subunits build the platform for the subsequent association with the DNA-binding subunit NF-YA [9]. The regulatory functions of NF-Y on the CCAAT-box have been mainly investigated for mammalian and yeast genes [1,3,4]. Each subunit is represented by a single gene in these organisms. Specificity and modulation of transcription activation is expected by interaction with other down-stream trans-acting factors [4].

In contrast, the plant NF-Y subunits are encoded by large gene families [10,11]. Initially 29 *Arabidopsis thaliana* genes encoding NF-YA, B or C subunits were presented [12], while subsequently a few additional genes were assigned to the NF-Y families comprising in total 10, 13 and 13 members of the NF-YA, NF-YB and NF-YC subfamilies, respectively [11]. Most of these genes are mainly characterized by diverse expression profiles in different tissues and during vegetative and generative development. The multiplicity of gene members in all three NF-Y families implies a complex role of plant NF-Y in transcriptional control. It has previously been proposed that the members of each gene family specifically contribute to activation of specific genes and the heterotrimeric NF-Y complex might act as combinatorial transcription factor. Thus, multiple genes could be regulated in space and time with a specific combination of different NF-Y subunits [12]. The combinatorial diversity of *Arabidopsis* NF-Y subunits was explored by interactome studies using the yeast-two-hybrid approach [13].

In heterogeneous NF-Y heterotrimers single subunits exhibit specific functions during transcriptional control, which could at least not entirely be substituted by another member of the same subunit family. This was demonstrated when a knock-out mutation of an individual *NF-Y* gene caused an obvious phenotype, e.g., during embryogenesis or stress adaptation. The *A. thaliana* mutants *leafy cotyledon 1 (lec1)* and *L1L* (*Lec 1-like*) showed a defective embryonic development [14,15]. The mutant genes belong to the *NF-YB* family and are described as essential regulators during morphogenesis and maturation of developing embryos. The role of LEC1 was defined for desiccation tolerance of seeds. Ectopic expression of *LEC 1* generates embryo-like structures on the leaf surface of transgenic *Arabidopsis thaliana* seedlings. Apart from this impact of NF-YB9 (LEC1) and NF-YB6 (LEC1-LIKE) other subunits were assigned to function in response to environmental stimuli or stress factors. NF-YA5 and NF-YB9 are required to control *LHCB1-3* genes in response to blue light and abscisic acid (ABA) [16]. The photoperiodically induced flowering-time was modulated in knock-out mutants for *NF-YB2* and *NF-YB3* genes and in the *nf-yc3/nf-yc4/nf-yc9* triple mutant under long day conditions [17,18]. It is proposed that a NF-Y complex bound to the promoter *of FLOWERING LOCUS T* (FT) is an important modulator of CONSTANS (CO)-mediated transcriptional activity of the *FT* gene [19].

Response to drought stress and endoplasmic reticulum (ER) stress has been reported to be mediated through NF-YA5 [20] and NF-YB1 [21] as well as NF-YA4, NF-YB3 and NF-YC2 [22]. Together with the transcription factor bZIP28, NF-Y binds to the *endoplasmic reticulum stress responsive element I* (ERSE-I) in combination with the CCAAT-box element [22].

Following the hypothesis that the differential expression pattern of each member of the three NF-Y gene families may indicate specific functions of single subunits, which cannot necessarily be replaced by other representatives of the same family [11], we aimed at evaluating the expression of *NF-YC* genes during adverse growth conditions, such as abiotic stress or herbicide treatments. A tobacco *NF-YC* gene was initially identified among early inducible genes upon accumulating porphyrin intermediates as a result of deregulated tetrapyrrole biosynthesis. Then, we intended to examine *Arabidopsis* genes homologous to this tobacco *NF-YC* gene. *Arabidopsis* T-DNA insertion mutants were selected and analyzed for phenotypic alterations during plant development and different oxidative stress conditions.

## 2. Results

### 2.1. Identification of Early Inducible Genes in Response to Photooxidative Stress Triggered by Accumulation of Coproporphyrin in Tobacco

**Figure 1:**
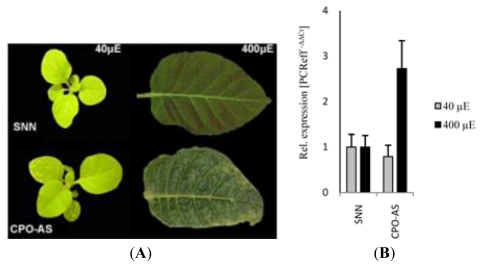
Tobacco *NF-YC2* is inducible early in *CPO* (coproporphyrinogen oxidase)-antisense plants. (**A**) *CPO*-antisense RNA expressing plants and control plants were grown under low light condition (left panels) before adult plants were transferred to high light. After exposure to high light, *CPO-*antisense plants show leaf necrosis; (**B**) Quantitative RT-PCR analysis of *NtNF-YC2* transcripts of CPO antisense and SNN wild-type plants before and 6 h after transfer from low (40 μM photon m^−2^·s^−1^) to high light intensities (400 μM photon m^−2^·s^−1^). (**A**) (**B**)

Transgenic tobacco lines expressing ectopically *CPO* (coproporphyrinogen oxidase) antisense RNA suffer from photodynamic cell death in leaf tissue [23]. CPO catalyzes the oxidative decarboxylation of two propionate side chains to vinyl groups of coproporphyrinogen leading to protoporphyrinogen. The necrotic cell death phenotype of *CPO*-antisense lines (Figure 1A) is explained by accumulation of the photoreactive CPO substrate coproporphyrinogen and the oxidized coproporphyrin. In continuation to the initial characterization of CPO-deficient tobacco plants we were interested to identify early inducible genes in response to coproporphyrin-induced cell death. We made use of a light-dose dependent phenotypical change of coproporphyrin-accumulating lines for the search of early inducible genes upon photooxidative stress. The leaf phenotype of wild-type and the *CPO-*antisense line #41 grown under short day condition (6 h light/18 h dark) at 200 μmol photon m^−2^·s^−1^ differed from that of plants exposed to long day condition (16 h light/8 h dark) at the same light intensity of 200 μmol photon m^−2^·s^−1^. Although, the plants already accumulate coproporphyrin under short day condition, leaf necrosis is not detectable. The shift from low light to a higher light dosage under the same light intensity led to visible cell death symptoms within 24–48 h (Figure 1A) [23,24].

To identify genes induced early under photooxidative stress conditions in the porphyrin-accumulating *CPO*-antisense tobacco plants, eight-week-old control and *CPO-*antisense plants (line 1/41) were grown under short day condition before the light exposure was extended for six additional hours and leaf samples were harvested for RNA extractions. Total RNA of control and wild-type plants was subjected to subtractive suppression hybridization. The approach resulted in the identification of, in total, 184 cDNA sequences with differential expression pattern between *CPO* antisense line 1/41 and wild-type plants, which encode 70 different proteins (Table S1). Sixty-one percent of the cDNA sequences were confirmed by reverse northern blotting to be expressed with elevated levels in the *CPO* antisense RNA expressing line.

Among the genes with increased expression in the transgenic line, *NtHAP5b* was identified as encoding the tobacco transcription factor subunit NF-YC. For consistency with the designation of the homologous *Arabidopsis* genes for the NF-Y subunits, we named this gene *NtNF-YC*. The induced *NF-YC* expression upon porphyrin-derived photooxidative stress was confirmed with quantitative reverse transcriptase polymerase chain reaction (qRT-PCR) analysis of tobacco wild-type and *CPO* antisense plants before and after transition to high light doses. The three-week-old plants were grown first under short day and low light condition (6 h light/18 h dark). Then control and transgenic plants were exposed six hours to low and high light before they were harvested for mRNA quantification (Figure 1B). *NtNF-YC* expression was induced during high light and the mRNA accumulated 4-fold compared to low light condition of control and CPO antisense seedlings as well as to high light-exposed wild-type seedlings. In conclusion, this screen enabled identification of a putative candidate gene encoding the C-subunit of the transcription factor NF-Y, which is inducible upon photoreactivity of accumulating tetrapyrrole intermediates.

### 2.2. Transcript Analysis of 36 Arabidopsis Genes Encoding NF-Y Subunits

**Figure 2:**
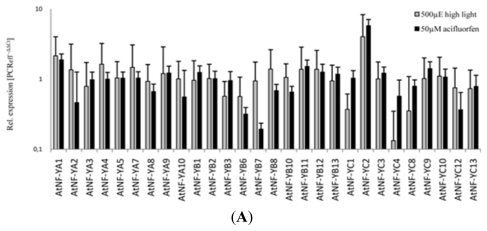

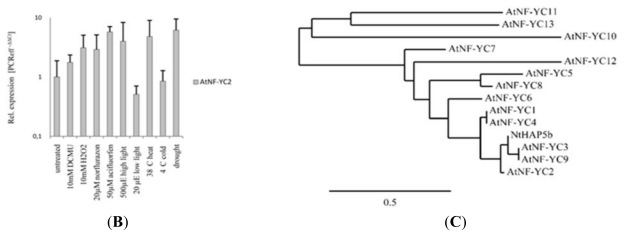
(**A**) Transcript profiles of *AtNF-Y* genes after acifluorfen treatment and high light exposure. Three-week-old *A. thaliana* (Col-0) seedlings were exposed to either 3 h of 500 μmol photon m^−2^·s^−1^ or treated with 50 μM acifluorfen for 3 h. The transcript levels were normalized to a member of the *SAND* gene family (At2g28390). RNA levels were assayed by qRT-PCR in comparison to those of control seedlings. Representatives of the 36 *Arabidopsis NF-Y* genes not listed in the graph were not detectable in seedlings. Only the *AtNF-YC2* transcript strongly accumulates after high light exposure and acifluorfen treatment; (**B**) *AtNF-YC2* transcript levels after exposure to various stress conditions and after treatment with herbicides. The *NF-YC2* RNA contents were compared in stress-exposed and control seedlings after normalization to transcript levels of the *SAND* gene. The different stress conditions and applications are described in Material and Methods. *AtNF-YC2* shows a strong accumulation of RNA in response to several adverse conditions; (**C**) Phylogenetic tree of selected NF-YC proteins. Full-length amino acid sequences of *Arabidopsis* NF-YC proteins and HAP5b (NtNF-YC) of *Nicotiana tabacum* were used for alignment (using MUSCLE 3.7 and Gblocks 0.91b), phylogenetic analysis (PhyML3.0 aLRT) and tree rendering (TreeDyn 198.3) performed with the phylogeny resource www.phylogeny.fr. [25].

Taking advantage of the previous exploitation of the entire *Arabidopsis* genome we searched for *NtNF-YC* homologs among the members of the *Arabidopsis NF-YC* gene family. To benefit from the complete collection of the *NF-Y* gene families for the three subunits we aimed at the identification of the candidate gene among the NF-YC family that is also induced upon porphyric stress. The highest similarity to NtNF-YC exists among a subgroup of the NF-YC family consisting of NF-YC1, NF-YC2, NF-YC3, NF-YC4 and NF-YC9 (Figure 2C*)*. These investigations for the identification of the *NtNF-YC*-homologous gene were embedded in a comprehensive analysis of the transcriptome of the complete set of all *NF-Y* genes in response to different adverse growth conditions including low temperature, heat, low and high light intensities and treatments of photodynamic herbicides, e.g., acifluorfen, norflurazon (inhibitor of carotenoid synthesis), DCMU (inhibitor of the linear photosynthetic electron transport chain), as well as oxidants, e.g., hydrogen peroxide. We quantified the transcript levels of all 36 *NF-Y* genes encoding the NF-YA, NF-YB and NF-YC subunits, which are currently known in *Arabidopsis* with qRT-PCR.

Table 1 surveys the results of the expression analyses and highlights those *NF-Y* genes which show a rapid and stronger change in expression (<0.5 or >2.0) (or >5.0 for drought stress) after the exposure to different stresses and effectors. The different stress conditions and applications are described in Material and Methods. Among the *NF-Y* genes only a few representatives of the *NF-YA, NF-YB* and *NF-YC* subfamilies reveal pronounced changes of their transcript levels upon application of several inducible stress conditions in comparison to normal growth condition. Drought stress and heat treatments lead to induction of more *NF-Y* genes, most likely because of the intensity of the stress (heat) and the extended stress period (drought). Among those genes with enhanced accumulation of mRNA content in response to stress, *NF-YA1* and *NF-YC2* responded more extensively with increased mRNA levels than other *NF-Y* genes.

In default of a coproporphyrin-accumulating *Arabidopsis cpo* mutant we applied acifluorfen to wild-type seedlings. Acifluorfen is a peroxidising herbicide that inhibits protoporphyrinogen oxidase, an enzyme of tetrapyrrole biosynthesis [27]. Figure 2A displays the transcript profile of all *NF-Y* genes 3 h after application of 50 μM acifluorfen and in a different experiment after 3 h of high light stress. Among these genes ultimately only *NF-YC2* accumulates six-fold higher RNA levels in response to acifluorfen treatment and high light stress (Figures 2A,B). Figure 2B depicts the transcript level of *NF-YC2* after multiple stress treatments indicating the strong oxidative stress inducibility. In summary, elevated *NF-YC2* transcript levels often correlate with oxidative stress (H_2_O_2_, heat, drought) or photooxidative stress (acifluorfen, norflurazon, high light), whereas *NF-YC2* mRNA amounts are slightly reduced under conditions which predominantly possess a reduced potential for oxidative stress (low temperature, low light). Among the *NF-YC* subfamily (Figure 2C) *NF-YC2* seems to have the strongest responsiveness against oxidative stress conditions. Enhanced *NF-YC2* expression upon porphyrins-induced photooxidative stress resembles the expression of *NtNF-YC* in *CPO*-antisense plants.

### 2.3. An Arabidopsis nf-yc2 Mutant and NF-YC2 Overexpressor Plants upon Oxidative Stress

**Figure 3:**
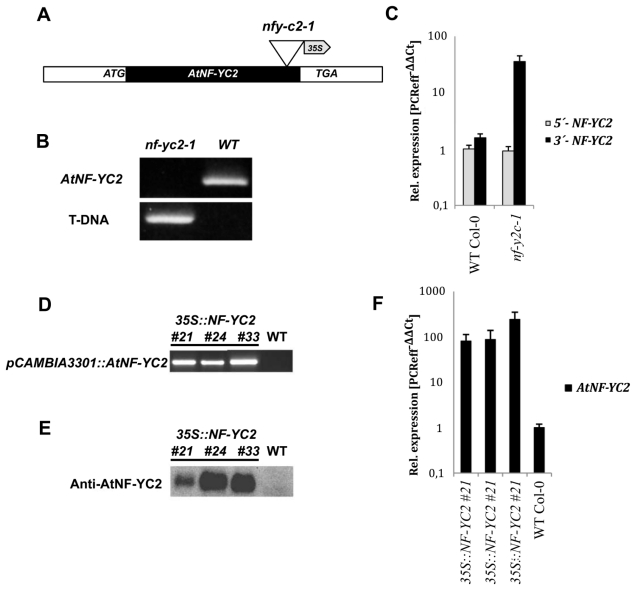
Analysis of the *Arabidopsis nf-yc2* mutant and *NF-YC2* overexpressor lines: (**A**) Schematic T-DNA-insertion site in *AtNF-YC2*; (**B**) Experimental confirmation of homozygosity of the T-DNA-inserted allele in *nf-yc2* in comparison to wild type. A PCR product of the endogenous *NF-YC2* gene could not be generated from DNA of *nf-yc2*; (**C**) The content of *NF-YC2* RNA in *nf-yc2* and control seedlings. The content of the *NF-YC2* RNA was determined with primer pairs upstream and downstream of the T-DNA insertion site in *NF-YC2*. A T-DNA-mediated stimulation leads to accumulation of 3′ transcripts in *nf-yc2* compared to control; (**D**) The *35SCaMV::AtNF-YC2* transgene was detected in the genomic DNA of the three representative transgenic lines #21, #24 and #33 in comparison to the wild-type genome; (**E**) Immunodetection of NF-YC2: Among the primary transformants the lines #21, #24 and #33 were selected to examine the *NF-YC2* expression. By means of anti-NF-YC2 antibodies the overproduction of NF-YC2 could be examined in plant extracts. The wild-type extract contains non-detectable amounts of NF-YC2; (**F**) Relative expression level of *AtNF-YC2* in wild-type and transgenic lines. Progenies of the T2 generation of the three transgenic lines were subjected to semi-quantitative RT PCR.

Among the potential T-DNA-insertion mutant lines for *NF-YC2* (SALK_11422; GABI_369E03, GABI_669A05) we identified only one mutant line with a T-DNA-insertion in the coding region (GABI_669A05). This line was designated *nf-yc2.* The exact T-DNA insertion site of *nf-yc2* could be confirmed 90 base pairs upstream of the translation stop codon (Figure 3A,B), while the inserted T-DNA of SALK_11422 and GABI_369E03 interrupted *NF-YC2* in the 5′ and 3′ untranslated region, respectively. The transcript levels of all T-DNA mutant lines were similar to wild-type seedlings (data not shown). But qRT-PCR revealed an increased content of the 3′ part of the cDNA downstream of the T-DNA-insertion site which can be explained with enhanced transcriptional activity due to promoter activity on the T-DNA (Figure 3C). It was expected that homozygous *nf-yc2* synthesizes a truncated protein. But the size, the amount and the stability of NF-YC2 in control plants and the homozygous *nf-yc2* mutant could not be determined. An antibody raised against a small non-conserved peptide sequence of NF-YC2 did not specifically recognize a protein of the expected size in the nuclear extract or total extracts of *Arabidopsis* wild-type seedlings. Efforts to enrich the specific antibody by purification attempts failed.

Seedlings of *nf-yc2* and wild type exposed to different abiotic stress conditions did not show differences of the macroscopic phenotype. This holds true, regardless, whether mutant and control plants were either treated with acifluorfen or Rose Bengal as well as exposed to high light or heat stress. When toxic amounts of herbicides were applied to the plants, formation of necrotic tissue upon acifluorfen treatment or bleaching in response to applied norflurazon were quantitatively similar in leaves of mutant and wild-type seedlings. We derived from these experiments that an obvious phenotypic diversification between *nf-yc2* and control seedlings could not be observed.

Additionally, more physiological properties of *nf-yc2* were examined in comparison to wild-type seedlings. Analyses of chlorophyll fluorescence parameters of mutant and wild-type seedlings revealed no significant differences in photosynthetic properties (Figure S1). Additionally, levels of tetrapyrrole intermediates were determined after a 36 h acifluorfen treatment. But the seedlings did not display significant differences in the content of tetrapyrrole intermediates (Figure S2). These results indicate that *nf-yc2* was not more sensitive towards photooxidative stress than control seedlings.

In order to examine the effect of ectopic *AtNF-YC2* overexpression on photooxidative stress response, the full-length cDNA sequence of *NF*-YC2 was inserted downstream of a CaMV 35S promoter in the pCAMBIA 3301 vector and introduced into the genome of *Arabidopsis thaliana* (Figure 3D). Selection of transformed lines with high expression of the transgene revealed lines #21, #24 and #33 with highly elevated *NF-YC2* mRNA levels (Figure 3F). Using the anti-NF-YC2 antibody, the overproduced protein was immunologically detectable in total cell extracts (Figure 3E), while the content of the endogenous NF-YC2 remained below the detection level in total wild-type extracts. The selected *NF-YC2* overexpressor lines were sprayed with 100 μM acifluorfen. However, no phenotypic differences were detectable on leaves of transgenic lines in comparison to wild-type leaves. Upon application of different concentrations of acifluorfen transgenic and wild-type seedlings showed always a similar formation of leaf necrosis after herbicide treatments. The viability of transgenic lines was also not elevated upon acifluorfen treatments in comparison to control seedlings after herbicide application (data not shown). Also the expression of the stress related *APX* genes (*APX1*, *APX2* and *APX4*), which are involved in the detoxification of reactive oxygen species (ROS) [28] and are induced by oxidative stress [29], were neither in *nf-yc2* nor in *35SCaMV::NF-YC2* significantly increased in relation to wild-type plants independent if acifluorfen were applied or not (data not shown).

### 2.4. Characterization of Other nf-yc Mutants Related to nf-yc2

In addition, the impact of photooxidative stress was also tested in other *Arabidopsis* T-DNA insertion mutants of the *Arabidopsis NF-YC* family with high sequence similarity to *NF-YC2* (Figure 3). T-DNA insertion mutants for the family members *AtNF-YC1* (GABI_004H11, SALK_86334), *AtNF-YC3* (GABI_51E10), *AtNF-YC4* (SALK_32163) and *AtNF-YC9* (SALK_058903) were isolated and homozygous lines were generated. With the exception of *nf-yc9,* homozygous *nf-yc1*, *nf-yc3* and *nf-yc4* mutant plants could always be identified with the T-DNA insertion in the coding region. In these T-DNA insertion lines no transcript of the respective *AtNF-YC* gene was detected (data not shown). *nf-yc3* (GABI_51E10) and *nf-yc4* (SALK_32163) as well as *nf-yc1-1* (GABI_004H11) and *nf-yc1-2* (SALK_86334) T-DNA insertion mutants neither showed phenotypically changes in response to treatments with acifluorfen nor to the exposure to high light. The growth of these mutants resembled that of wild-type and *nf-yc2* seedlings.

### 2.5. Modified Flowering Time of NF-YC1 and NF-YC2 Overexpressor Lines

Apart from the transcript analysis and the assessment of phenotypic differences between mutant and control seedlings upon abiotic stress, *NF-YC2* overexpressor lines and the T-DNA insertion mutant *nf-yc2* were examined for induction of flowering under short and long day conditions. In parallel, *nf-yc1-2* as well as *AtNF-YC1* overexpressor lines were also tested as representatives of the *NF-YC* subfamily with high homology to NF-YC2. Genotyping of *nf-yc1-2* revealed the T-DNA insertion in the exon region (Figure S3A). Homozygous *nf-yc1-2* does not contain detectable amounts of *NF-YC1* transcripts (Figure S3B). *35SCaMV::NF-YC1* expression in transgenic *Arabidopsis* lines yielded a strong accumulation of the specific mRNA. The lines #41, #42 and #57 represent transformants with over-expression of *NF-YC1* (Figure S3C,D).

**Figure 4:**
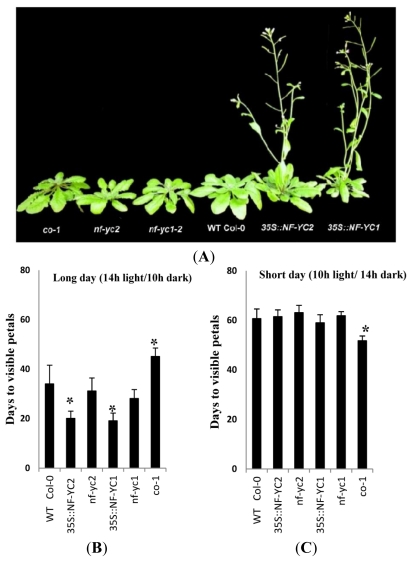
(**A**) Developmental stage and flowering phenotype of *Arabidopsis* wild-type (Col-0), *nf-yc2, nf-yc1-2*, *co-1* and representatives of transgenic lines overexpressing ectopically *NF-YC2* or *NF-YC1* 38 days after germination. Expression of *35S::NF-YC1* and *35S::NF-YC2* leads to induction of early flowering under long day condition in comparison to wild-type plants (**B**). Time period between germination and development of first visible petals is indicated under long day (**B**) or short day condition (**C**).

Thirty-eight-day-old seedlings of the transgenic lines 35SCaMV::NF-YC1 #41, #42, #57 and 35SCaMV::NF-YC2 #21, #24 and #33 showed a significant early flowering phenotype under long day condition compared to wild-type plants and *nf-yc2* and *nf-yc1-2*. Figure 4A shows always one representative plant of each set of overexpressor lines. The overexpressor lines flowered at least 16 days earlier than wild type (Figure 4B). Under short day condition all mutants and overexpressor lines did not significantly modulate the flowering time (Figure 4C). Flowering was set when petals started to be seen during flower development. As a control, *co-1*, a mutant of *CONSTANS* (*co-1*) showed a significantly delayed flowering than wild-type plants under long day condition (Figure 4B). In consistencies with a previous report *co-1* flowered significantly earlier under short day condition (Figure 4C) [30].

### 2.6. Modified Expression of Genes Involved in Flowering Induction in 35SCaMV:NF-YC2 and 35SCaMV:NF-YC1 Overexpressor Lines

**Figure 5:**
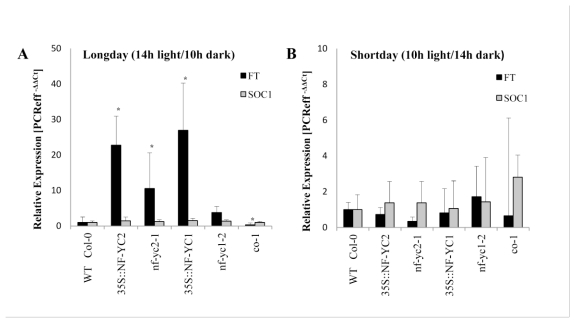
Relative expression profiles of *FLOWERING LOCUS T* (*FT*) and *SUPRESSOR OF CONSTANS1* (*SOC1*) in *Arabidopsis* wild-type (Col-0), *nf-yc2, nf-yc1-2* and a representative line each of *NF-YC2* and *NF-YC1* overexpressor plants. RNA was isolated in plants grown under long day (**A**) and short day condition (**B**). The leaf material was harvested before the expected induction of flowering. Quantitative RT-PCR revealed increased *FT* RNA contents in *NF-YC2* and *NF-YC1* overexpressing lines relative to wild-type RNA. In each RNA sample *FT* and *SOC1* RNA were normalized to constitutive expression of *SAND* (At2g28390).

In 21-day-old mutant seedlings with modified expression of *NF-YC1* and *NF-YC2* we explored transcript levels of *FLOWERING LOCUS (FT)* and *SUPRESSOR OF OVEREXPRESSION OF CO1 (SOC1)* [31], two genes in the photoperiodic control of flowering by qRT-PCR. Growth under long day condition (14 h light/10 h dark) leads to a 20-fold accumulation of *FT* transcripts in the earlier flowering periods of the *35SCaMV::NF-YC1* and *35SCaMV::NF-YC2* lines (Figure 5A). The two mutants *nf-yc1-2* and *nf-yc2* also showed elevated *FT* RNA levels, which do not correlate with a significant variation in flowering time in comparison to wild-type properties. As control *co-1* contained lower *FT* mRNA levels under long day condition, which correlate with the late flowering phenotype (Figure 5A). Short day exposure (10 h light/14 h dark) of the *NF-YC1* and *NF-YC2* overexpressor lines did not result in modified expression of *FT* (Figure 5B). The *SOC*1 transcript levels were not modulated under long or short day condition in overexpressor plants and T-DNA insertion mutants in comparison to wild-type plants (Figure 5A,B).

## 3. Discussion

Among the members of the *Arabidopsis NF-YC* family, only *NF-YC2* shows an induced expression under oxidative stress conditions and is a candidate for the transcriptional activation in response to oxidative stress. Besides its inducibility upon several oxidative stress conditions, *AtNF-YC2* expression was drastically enhanced after acifluorfen treatment. The inactivation of protoporphyrinogen oxidase leads to accumulation of the photoreactive protoporphyrinogen, which, upon light exposure, is autooxidized to protoporphyrin and generates reactive oxygen species. The photodynamic stress generated by acifluorfen treatment resembles the photosensitization of tobacco *CPO*-antisense RNA-expressing plants under high light conditions [23,24]. Furthermore, *AtNF-YC2* shares the responsiveness to photooxidative stress with the tobacco homologous *NtNF-YC* gene (Figure 1). The tobacco NF-YC subunit belongs to the same phylogenetic subclade as the *A. thaliana* proteins NF-YC1, 2, 3, 4 and 9 (Figure 2C), but among the *Arabidopsis NF-YC* subfamily NF-YC2 does not show the highest similarity to NtNF-YC.

Porphyrin-accumulating transgenic plants as well as acifluorfen-treated plants trigger a necrotic cell death phenotype indicating a mechanism that integrates accumulation of photooxidative stress during a time period of light exposure. It is suggested that the reactive oxygen species generated during these photodynamic processes might induce the *NF-YC2* expression. The elucidation of additional roles of both homologous representatives of NF-YC subunits in both plant species requires further investigations. Apart from the stress-induced *NtNF-YC* it remains also elusive, how many additional genes belong to the *NF-YC* family in *Nicotiana tabacum*.

Despite of the clear induction pattern of *AtNF-YC2* and its tobacco *NF-YC* homolog, the *Arabidopsis nf-yc2* mutant and the *NF-YC2* overexpressor seedlings do not show phenotypic modulation in their response to (photo)oxidative stress. Derived from the expression data *nf-yc2* mutants can most likely still express a truncated protein which lacks part of the transcription activation domain of NF-YC but still possesses the histone-fold motif. It is not excluded that the truncated NF-YC2 still contributes to the assembly of intact NF-YC heterotrimers. But also NF-YC2 overexpressors did not show a positive or adverse effect upon exposure to oxidative stress. These missing phenotypical alterations in response to oxidative stress are likely explained with the redundancy among NF-YC subunits. When expression of one *NF-YC* gene is depleted, another member of the family might compensate the deficiency. The compensatory capacities among transcription factor families have been proposed in the literature [32]. Neither mutants for *NF-YC1*, *NF-YC3* and *NF-YC4* nor *nf-yc1/nf-yc2* double mutants (data not shown) or *NF-YC1* overexpressor mutants showed an impaired response upon or increased sensitivity to oxidative stress.

Instead of phenotypical changes during oxidative stress, *NF-YC2* and *NF-YC1* overexpressors displayed early flowering during long day condition (Figure 4). This is correlative with the increased *FT* expression in the overexpressor lines (Figure 5). It is suggested that NF-YC2 and NF-YC1 contribute to the transcriptional control of *FT*. Along these lines *co-1* mutants show an extended flowering time during long day condition which correlates with strong reduction of *FT* expression (Figures 4 and 5) [30].

Day length dependent flowering in *Arabidopsis* is predominantly regulated by two transcription factors CO and FT. Under long day condition CO activates transcription of *FT* in the leaves, whereas the FT protein is transported as phloem mobile flowering signal to the shoot apex. In the apical shoot meristem, FT together and another transcription factor FLOWERING LOCUS D (FD) are responsible for induction of *SOC1* and *APETALA 1* (AP1) encoding two major regulators of the conversion into the inflorescence meristem [33].

The current model of transcriptional control of *FT* includes the function of CO as major regulator via a specific *cis* element inside the *FT* promoter, also described as CONSTANS responsive element (CORE) [19]. The *Arabidopsis* NF-Y complex is assumed to act as modulator of the CO-mediated transcription activation of *FT*. As a potential recruitment site a CCAAT-box inside the *FT* promoter was hypothesized [17], which is not responsible for the transcription activation by CO [19]. Nevertheless diverse NF-Y proteins physically interact with CO and other representatives of the CO-like family [34,35].Therefore, both transcription factors, CO and NF-Y, are suggested to mutually influence each other in their function on the *FT*-promoter.

The exact molecular function of NF-Y proteins during the day length-dependent flowering induction remains still unclear. Down-stream contribution of NF-Ys in other flower-inducing pathways are not excluded. In this context it is worth to be mentioned that gibberellins (GAs) play an important role during the conversion of vegetative to floral tissue at the apical shoot meristem. GAs are embedded in different pathways influencing flower induction in *Arabidopsis* [36]. On the one hand GAs promote flowering independent from the CO/FT-mediated regulatory mechanism under short day conditions in a non-photoperiodic manner [37]. On the other hand GA-mediated effects on flowering time can be observed under short and long day conditions [38]. Moreover, a GA-dependent mechanism regulating the expression of *FT* cannot be excluded [36], especially due to the fact that GAs application can result in increased *FT* transcript levels and early flowering under long day conditions in *Arabidopsis* [39].

The physiological relevance of NF-Y during photoperiodic flower induction became obvious by investigation of several *nf-y* mutants showing modified flowering time. This clear phenotype can be contributed to the mutants *nf-yb2* and *nf-yb3* as well as the *nf-yb2/b3* double and *nf-yc3/c4/c9* triple mutants [17,18]. While the deficit of one or several of the above mentioned NF-Y subunits leads to a delayed flowering phenotype under long day conditions, overexpressing of *NF-YB2* or *NF-YB3* yielded in an early flowering phenotype under identical conditions. On the other hand no effect can be observed in these overexpressor lines in short day light periods [17].

The early flowering phenotypes of both *NF-YC2* and *NF-YC1*-overexpressing mutants underline a similar function of NF-YC2 and NF-YC1 in day length depending floral induction. Similar to *NF-YC3*, *NF-YC4* and NF-Y*C9*, single knock-out mutation of *NF-YC2* or *NF-YC1* did not lead to phenotypical changes in flower induction. While the effect of NF-YC3, C4 and C9 on flower induction could only be observed by simultaneous knock out of all three subunits in the *nf-yc3/nf-yc4/nf-yc9* triple mutant [18], *nf-yc1/nf-yc2* double mutant plants did not show a similar phenotype (data not shown).

## 4. Material and Methods

### 4.1. Growth of Plants

*Arabidopsis thaliana* plants grew for 14, 21 or 34 days under light/dark conditions (120 μM photons m^−2^·s^−1^, 12 h light/ 12 h dark at 23 °C) before they were exposed to high light (HL—500 μM photons m^−2^·s^−1^), low light (LL—50 μM photons m^−2^·s^−1^), increased (38 °C) or low temperature (4 °C) respectively. Alternatively, plants were treated with 50 μM acifluorfen, 10 mM DCMU, 10 mM H_2_O_2_ or 20 μM norflurazon by spraying leaves with these agents. Leaf material was harvested after exposure time of 3 h. Drought stress was induced when plants were not watered for 8 days.

For RNA quantification *Nicotiana tabacum* var. Samsun NN (SNN) and *CPO*-antisense plants [23] were initially cultured for 14 days under low light conditions (40 μM photons m^−2^·s^−1^, 12 h light/ 12 h dark at 27 °C) before exposure to high light (400 μM photons m^−2^·s^−1^, 12 h light/ 12 h dark at 27 °C). Leaf material was harvested before and 6 h after exposure to high light. For *suppression subtractive hybridization* experiments tobacco plants were cultivated for 8 weeks with a low light dose (200 μM photons m^−2^·s^−1^, 6 h light/ 18 h dark at 25 °C) and were subsequently exposed to a high light dose (400 μM photons m^−2^·s^−1^) for 12 h before leaf material was harvested.

### 4.2. RNA—Quantification

For qRT-PCR total RNA of 100 mg leaf material was extracted with RNeasy mini kit (Qiagen, Germany) in accordance to the supplier’s manual. The amount of 1.250 ng of total RNA were treated with 2U TURBO DNA-*free* (Ambion, USA) for removing genomic DNA contaminations before first strand cDNA synthesis was performed using SuperScript III reverse transcriptase (Invitrogen, USA) in the presence of 50 ng oligo dT_18_-primer (Sigma, Germany). As a template for qRT-PCR analysis ten times diluted cDNA were used in 20 μL reactions (1 μL cDNA, 500 nM each primer, 0.4 μL 50 × SYBR Green and 10 μL ImmoMix TM (Bioline, Germany) performed with the Rotor Gene 2000 (Corbert-Research, Australia) or 5 μL PCR reactions (0.5 μL cDNA, 200 nM each primer and 2.5 μL SYBR Green PCR Master Mix (Applied Biosystems, USA) performed in 384 wells plates using the ABI PRISM 7900HT sequence detection system (Applied Biosystems, USA). The amplification program consisted of an initial hot-start activation at 94 °C for 10 min, 45 cycles including 15 s denaturing at 94 °C, 20 s annealing at 60 °C and 20 s elongation phase at 72 °C. Primers of the *Arabidopsis thaliana NF-Y* gene families were kindly provided by the Max-Planck Institute of Molecular Plant Physiology Golm. Oligonucleotide sequences are listed in the Table S2. Transcript levels of each gene were normalized to an *Arabidopsis* member of the *Arabidopsis SAND* gene family (At2g28390) or *Nicotiana tabacum ACTIN* gene respectively. Primer efficiency was determined by linear regression on the Log(fluorescence) of each PCR reaction using the LinRegPCR software [40] and PCR efficiency was defined as mean value for each primer pair. For semi-quantitative RT-PCR, total RNA were isolated with TRIsure (Bioline, Germany) and treated with 1U DNAseI (Fermentas, Germany). First strand cDNA synthesis was performed by RevertAid M-MuLV reverse transcriptase (Fermentas, Germany) in accordance to the manufacturer instructions.

### 4.3. Plant Mutants

Full length cDNA constructs of *AtNF-YC1* and *AtNF-YC2* were amplified by PCR using specific primers (AtNF-YC1: 5′-ACCATGGATACCAACAACCAGC-3′ and 5′-TGACGTCCACCTTGGCCG TCGAGA-3′; AtNF-YC2: 5′-ACCATGGAGCAGTCAGAAGAGG-3′ and 5′-TGACGTCCAGACTC ATCAGGGTGTTG-3′) and ligated into NcoI and ActII restriction sites of the multiple cloning site of the modified pCAMBIA3301 plasmid. The correct sequence of the *35SCaMV::AtNF-YC1* and *35SCaMV:: AtNF-YC2* gene constructs was confirmed by sequencing and used for *A. tumefaciens-*mediated stable transformation of *Arabidopsis* (ectoype Col-0) plants by means of the floral-dip transformation method described previously [41]. Transformed plants were selected by successive treatments of two, three and four-week-old seedlings with 0.1% (w:v) BASTA (Bayer CropScience AG, Germany). T-DNA insertion mutants of the SALK and GABI-Kat collection were obtained from the European Arabidopsis Stock Centre (Nottingham, UK) or Bielefeld University, Germany. Homozygous T-DNA insertions were validated by PCR using specific oligo primer for either the wild-type allele or the T-DNA left boarder and for indication of different genes (primer sequences in Table S2). Transcript levels of the affected genes in mutant plants were analyzed as described above. AtNF-YC2 protein amounts of *pCAMBIA3301::AtNF-YC2* plants were determined by immunoblot analysis in accordance to standard methods [42].

### 4.4. Chlorophyll Fluorescence Analyses

*In vivo* chlorophyll fluorescence was measured with attached leaves of dark-adapted plants as previously described [43]. The following fluorescence parameters were assessed: the maximum photochemical efficiency of PS II in the dark-adapted state *F*_v_/*F*_m_ = (*F*_m_ – *F*_0_)/*F*_m_, effective quantum yield of photochemical energy conversion in PSII (*ΦP*_SII_ = (*F*_m_′ – *F*_t_) *F*_m_′^−1^) and non-photochemical quenching (NPQ = (*F*_m_ – *F*_m_′) *F*_m_′^−1^ ) in accordance to [44]. Photon flux densities were measured using a quantum sensor (LI-189A, Li-Cor, Lincoln, NE).

### 4.5. Analysis of Porphyrins

Extraction and analysis of accumulated tetrapyrroles in tobacco leaves were essentially performed as described [23]. Modifications regard the extraction of liquid nitrogen-ground leaf material with 50 mM potassium phosphate buffer (pH 7.8), methano1:0.l M NH_4_OH (9:1, *v*/*v*) and acetone: 0.l M NH_4_OH (9:1, *v*/*v*). The porphyrins were separated by HPLC (Agilent, Germany) on a RP 18 column (Novapak C18, 4 μm particle size, 4.6 × 250 mm). Column eluent was monitored by fluorescence (λ_ex_ 405 nm, λ_em_ 625 nm) and porphyrins were identified and quantified by authentic standards.

### 4.6. Suppression Subtractive Hybridization

Two μg polyA^+^ RNA of the sixth tobacco leaf (counting from the top of the plant) from wild-type (driver) and CPO antisense # 41 (tester) plants was extracted and applied to the forward subtracted and the reverse subtracted approach according to the protocol of the supplier (Clontech, Germany). After *E. coli* transformation with pCRII vector (Invitrogen, USA) containing forward subtracted cDNA fragments 1993 colonies were obtained, which were subjected to a colony hybridization using the labeled probes from forward and reverse subtracted cDNA. Finally, 234 bacterial colonies were confirmed and their cDNA sequenced.

### 4.7. Statistical Analysis

All data shown in graphs are mean values and corresponding standard deviations are displayed as error bars calculated using the Excel 2007 software (Microsoft Corp., USA). For relative expression analysis three biological replicates were used, whereas fluorescence parameter, tetrapyrrole intermediates and days to visible petals were determined by evaluation of 12 biological replicates (different plants). Differences between WT and mutant lines were tested using a two-tailed Student’s *t test* and were regarded as significant for *p* < 0.05.

## 5. Conclusion

As a conclusion, it seems to be reasonable that the subunits NF-YC1, NF-YC2, NF-YC3, NF-YC4 and NF-YC9 are involved in the control of day length dependent floral induction. These five members of the *Arabidopsis* NF-YC family can be clustered in one phylogenetic branch possessing a high similarity of their amino acid sequence. The elucidation of specific roles of the individual NF-YC subunits remains a challenging task, due to the obvious overlap of their functional properties.

## Figures and Tables

**Table 1:** Overview of AtNF-Y genes with altered expression in response to abiotic stress. AtNF-Y genes revealing relative expression of >2.0 (>5.0 at drought stress) or <0.5 are listed in combination with the determined values. Lower and upper limit of the confidence interval in accordance with [26] appear in parentheses. Growth conditions under stress treatment are described in Material and Methods.

Stress condition	*AtNF-*Y with increased expression		*AtNF-Y* with lower expression	

	*AtNF-Y*	relative expression	*AtNF-Y*	relative expression
10 mM H_2_O_2_	*ATNF-YA1*	5.0 (1.6–15.8)	*ATNF-YB7*	0.3 (0.2–0.7)
	*ATNF-YC2*	3.1 (1.9–5.1)	*ATNF-YC12*	0.5 (0.4–0.7)

20 μM Norflurazon	*ATNF-YA1*	2.3 (1.3–3.9)	*ATNF-YB1*	0.4 (0.2–0.7)
	*ATNF-YA10*	3.1 (2.0–4.9)	*ATNF-YB6*	0.4 (0.2–0.6)
	*ATNF-YC2*	2.9 (1.7–5.1)	*ATNF-YC12*	0.5 (0.3–0.8)

50 μM Acifluorfen	*ATNF-YC2*	5.8 (4.7–7.1)	*ATNF-YA2*	0.5 (0.2–1.3)
			*ATNF-YB6*	0.3 (0.3–0.4)
			*ATNF-YB7*	0.2 (0.2–0.2)
			*ATNF-YC12*	0.4 (0.2–0.6)

High light 500 μE	*ATNF-YA1*	2.1 (1.1–4.0)	*ATNF-YC1*	0.4 (0.2–0.6)
	*ATNF-YC2*	4.0 (1.9–8.3)	*ATNF-YC4*	0.1 (0.1–0.3)
			*ATNF-YC8*	0.4 (0.1–1.1)

Low light 20 μE	*ATNF-YC4*	2.1 (1.2–3.5)	*ATNF-YC2*	0.5 (0.4–0.7)
			*ATNF-YC13*	0.4 (0.3–0.5)

Heat stress 38 °C	*ATNF-YA1*	4.2 (2.6–6.8)	*ATNF-YB7*	0.3 (0.2–0.5)
	*ATNF-YA5*	2.5 (1.7–3.7)	*ATNF-YC13*	0.1 (0.0–0.1)
	*ATNF-YA7*	2.3 (1.2–4.6)		
	*ATNF-YB13*	2.5 (1.4–4.4)		
	*ATNF-YB6*	3.6 (1.9–6.6)		
	*ATNF-YB8*	4.1 (2.7–6.3)		
	*ATNF-YC10*	2.1 (1.3–3.3)		
	*ATNF-YC2*	4.8 (2.5–9.0)		
	*ATNF-YC4*	9.4 (6.6–134)		

Cold stress 4 °C			*ATNF-YB2*	0.4 (0.3–0.5)
			*ATNF-YB3*	0.2 (0.2–0.4)
			*ATNF-YB8*	0.4 (0.2–0.7)
			*ATNF-YC12*	0.4 (0.2–0.6)
			*ATNF-YC4*	0.4 (0.2–0.6)

Drought stress	*ATNF-YA1*	21.7 (7.3–64.6)	*ATNF-YC1*	0.4 (0.2–0.8)
	*ATNF-YA10*	48.0 (6.7–345.6)		
	*ATNF-YA3*	5.9 (3.1–11.4)		
	*ATNF-YA4*	5.4 (3.6–8.1)		
	*ATNF-YA5*	9.7 (4.1–23.1)		
	*ATNF-YA7*	7.9 (5.4–11.5)		
	*ATNF-YB1*	9.0 (5.0–16.0)		
	*ATNF-YB10*	22.6 (8.9–57.7)		
	*ATNF-YB6*	57.4 (30.7–107.3)		
	*ATNF-YB7*	5.2 (2.8–9.5)		
	*ATNF-YC10*	15.2 (9.0–25.8)		
	*ATNF-YC2*	6.2 (4.0–9.5)		
	*ATNF-YC3*	7.6 (4.3–13.4)		
	*ATNF-YC4*	5.7 (3.3–10.2)		
